# Albuminuria as a non-invasive biomarker of endothelial dysfunction in patients with COPD

**DOI:** 10.1038/s41598-025-32462-4

**Published:** 2026-01-06

**Authors:** Marwa Moaaz, Sahar Mourad, Ayman Baess, Rania Sweed

**Affiliations:** 1 Department of Human Physiology, Clinical Respiratory Physiology Unit, Medical Research Institute, Alexandria University, Alexandria, Egypt; 2 Department of Chest Diseases, Faculty of Medicine, Alexandria University, Alexandria, Egypt

**Keywords:** Chronic obstructive pulmonary disease, Albuminuria, Endothelial dysfunction, Hypoxemia., Physiology, Pathogenesis

## Abstract

Endothelial dysfunction (ED) plays a significant role in the pathogenesis of chronic obstructive pulmonary disease (COPD). While albuminuria is currently recognized as a biomarker of generalized ED, data on the evaluation of albuminuria among COPD patients and its association with disease outcome measures are still limited. Thus, the aim of this study was to assess albuminuria among a group of COPD patients and investigate its relationship with clinical and physiological parameters. Sixty adult patients with COPD and forty non-COPD adult smokers were included in this cross-sectional study. All participants were assessed for anthropometric parameters, oxygen saturation (SpO_2_), spirometry test, 6-minute walk test, flow-mediated dilation (FMD) of the brachial artery, routine laboratory measurements, and urinary albumin-to-creatinine ratio (UACR). Patients with COPD had higher levels of UACR (mg/g) and a greater prevalence of albuminuria than non-COPD smokers. COPD patients with albuminuria had higher body mass index (BMI), more frequent exacerbations, lower SpO_2_, lower FMD, and higher fasting plasma glucose than patients without albuminuria. UACR in COPD patients was associated negatively with FMD and SpO_2_ and positively with the number of comorbidities and fasting plasma glucose. The main predictors of albuminuria in COPD patients were high BMI, low SpO_2_, and high fasting plasma glucose. The only independent predictor of albuminuria was the presence of low SpO_2_. Given the significant association with FMD, the gold standard measure of ED, we suggest that the measurement of UACR could be routinely utilized for assessing ED in COPD patients, particularly those with low oxygen saturation.

## Introduction

Chronic obstructive pulmonary disease (COPD) is a worldwide health problem and a leading cause of mortality. It represents a heterogeneous spectrum of lung pathologies primarily induced by prolonged inhalational exposures. The four key pathologic processes in COPD include small airway disease, mucus hypersecretion, emphysema, and pulmonary vascular dysfunction^1^.

The pulmonary vascular dysfunction in COPD, especially in the vascular phenotype, is a component of the generalized vascular endothelial dysfunction (ED) that develops in response to inhaled insults. ED plays a significant role in the natural history of COPD and contributes to the pathogenesis of many comorbidities, including cardiovascular disease (CVD), the most prevalent comorbidity in COPD patients that results in the worst disease outcomes^2^. This indicates the need for early and regular evaluation of endothelial function in COPD patients to ensure early detection and rapid intervention.

Assessing endothelial function is complex, and while numerous techniques have advanced research in this area, none have been accepted as diagnostic tools in routine clinical practice. This may result from their invasive nature or their requirement for patient preparation and cooperation, which may be demanding for some patients^3^. In clinical settings, the ultrasound-based assessment of flow-mediated dilation (FMD) of the brachial artery has been regarded as the reference procedure for evaluating endothelial function. Nevertheless, its use in daily clinical practice can be challenging considering the need for precise standardization, adherence to strict protocols, experienced operators, and a controlled environment^4^. In addition, evidence exists that ED in COPD patients occurs not only in the macrovasculature, assessed by FMD, but also in the microvasculature. This suggests that other methods capable of evaluating the microvascular function should be employed to assess ED in COPD patients alongside FMD, as they evaluate various components of vascular biology^3^.

Several biomarkers were proposed to assess the microvascular endothelial function in individuals who are at high risk for CVD, yet just a few of them have gained acceptance for clinical use. From this perspective, albuminuria, traditionally regarded as an indicator of renal dysfunction, was recently recognized as a biomarker of generalized ED and was strongly correlated with the direct measures of microvascular endothelial function in the skin and retina^5,6^. In addition, several guidelines recommended screening for albuminuria, using the urine albumin-to-creatinine ratio (UACR), for evaluation of vascular function in individuals at high risk^7^. Being affordable, noninvasive, and easily measurable, this could serve as a promising biomarker for early identification of ED in COPD patients.

However, despite the established role of ED in the pathogenesis of COPD and its comorbidities and the recognition of albuminuria as a biomarker of systemic vascular ED, data regarding the evaluation of albuminuria in COPD patients and its correlation with disease outcomes remain limited. Accordingly, we performed this observational cross-sectional study to assess albuminuria in a group of adult patients with stable COPD and investigate its associations with clinical and physiological parameters.

## Materials and methods

### Study subjects

This study was conducted on a random sample of current and ex-smokers attending the chest outpatient clinic of Alexandria Main University Hospital (AMUH), Alexandria, Egypt. The study procedures were performed at the Clinical Respiratory Physiology Unit, Medical Research Institute, Alexandria University, Egypt. The participants were chosen according to the predetermined inclusion and exclusion criteria. They were allocated to one of the two study groups according to the presence or absence of the Global Initiative for Chronic Obstructive Lung Disease (GOLD) criteria for diagnosis of COPD^8^.

The patient group included sixty adult patients. Their inclusion criteria were an established diagnosis of COPD according to the GOLD criteria^8^, age ≥ 40 years, and having a stable disease (free of exacerbation) for ≥ 4 weeks before the start of the study. The exclusion criteria were the presence of acute or chronic respiratory disease other than COPD, an established diagnosis of hypertension, diabetes mellitus, or chronic kidney disease, and pregnancy or breastfeeding for female participants. The control group included forty age- and sex‐matched adult smokers with the same exclusion criteria, in addition to being free of COPD.

Participants were invited to freely volunteer for the study, and informed written consent was obtained before inclusion. The study protocol was approved by the Ethics Committee of the Faculty of Medicine, Alexandria University, Egypt (approval no. 0106712).

### Methods

#### Assessment of personal data and anthropometric and clinical parameters

All subjects underwent detailed personal history taking, including age, residence, and smoking history. The pack-year index was calculated as the number of packs of cigarettes smoked per day x the smoking duration in years^9^. Patients with COPD reported a complete medical history, including the history of acute exacerbations during the previous year and hospitalization. The history of comorbidities was obtained, and the Charlson Comorbidity Index (CCI) was calculated^10^. The modified Medical Research Council (mMRC) scale^8^, COPD assessment test (CAT™)^11^, and St. George’s respiratory questionnaire (SGRQ)^12^ were used to assess different aspects of respiratory symptoms. Finally, the severity of COPD was graded according to the GOLD combined assessment tool^8^.

Subsequently, all participants were subjected to a complete general and local chest examination, including assessment of heart rate, arterial blood pressure, and peripheral oxygen saturation (SpO_2_) at rest. The SpO_2_ was measured at the fingertip using a pulse oximeter. The anthropometric measurements included weight (Kg), height (cm), and waist circumference (cm), and the body mass index (BMI) was calculated as [weight (kg)/square of height (m^2^)].

#### Physiological assessment

##### Spirometry

A standardized baseline spirometry test was performed according to the American Thoracic Society (ATS)/ European Respiratory Society (ERS) guidelines^13^, using a MasterScreen™ Pneumo Spirometer (CareFusion, Hoechberg, Germany) with a calibrated Jaeger™ pneumotachograph. Every participant was trained to perform the test correctly and completed at least three acceptable and repeatable trials, and the highest value was recorded as a percentage of predicted value (%pred). Forced expiratory volume in one second (FEV_1_), forced vital capacity (FVC), the ratio between them (FEV_1_/FVC%), peak expiratory flow (PEF), and forced expiratory flow at 75% and 25–75% of FVC (FEF_75%_ and FEF_25−75%_, respectively) were measured. Finally, for COPD patients, a bronchodilator responsiveness test was performed according to the ATS/ERS guidelines^13^.

##### Six-minute walk test

The six-minute walk test (6MWT) was also performed according to the ERS/ATS guidelines^14^. At the end of the test, the six-minute walk distance (6MWD) was recorded as the total distance covered to the nearest meter. The BODE index^15^ was calculated using the BMI (kg/m^2^), airflow obstruction assessed as the post-bronchodilator FEV_1_ (%pred), the degree of dyspnea assessed by the mMRC dyspnea scale, and exercise performance expressed as the 6MWD (m).

##### Flow-mediated dilation of the brachial artery

Thereafter, the FMD of the brachial artery was assessed using a standardized protocol consistent with the European Society of Cardiology Consensus^16^. The brachial artery baseline diameter was measured using high-resolution B-mode ultrasound with a 15-MHz linear array transducer (SONOS 5500; Philips, Andover, MA). A blood pressure cuff was inflated to occlude arterial flow to the forearm, then the post-deflation diameter was monitored until the true peak diameter was successfully captured. FMD was calculated as the percentage of change in the artery diameter from baseline.

#### Laboratory assessment

Laboratory investigations included serum creatinine, fasting blood glucose, and total serum cholesterol levels. The glomerular filtration rate (GFR) was estimated using the Chronic Kidney Disease Epidemiology Collaboration (CKD-EPI) equation^17^.

##### Urinary albumin excretion

Finally, a first-voided clean-catch morning urine sample^18^ was analyzed for its albumin (mg/dL) and creatinine (g/dL) concentrations. Urine albumin concentration was determined by a standard immunoturbidimetric method using an automated Cobas-C Roche^®^ analyzer. Urine creatinine concentration was determined using Jaffe’s reaction and quantified using the Cobas-C Roche^®^ system. The urinary albumin-to-creatinine ratio (UACR) was reported as [urine albumin (mg/dL)/ urine creatinine (g/dL)]. The presence of albuminuria was defined as the UACR being ≥ 20 mg/g in our male patients^19–21^.

### Statistical analysis

Data were analyzed using the IBM SPSS software package version 20.0. (Armonk, NY: IBM Corp). The categorical data were represented as numbers and percentages [number (percentage)]. The chi-square test (χ^2^) was applied to compare two groups. Alternatively, either the Monte Carlo or the Fisher Exact correction test was used when more than 20% of the cells had an expected count of less than five.

The distribution of continuous data was tested for normality using the Shapiro-Wilk test. Continuous quantitative data were expressed as mean and standard deviation (mean ± SD) if they proved to be normally distributed, or median and interquartile range [median (IQR)] if not normally distributed. Student t-test was used to compare the two studied groups for normally distributed quantitative variables, and the Mann-Whitney test was used to compare the two groups for not normally distributed quantitative variables. The significance of the results obtained was judged at the 5% level.

The degrees of association between the UACR and other parameters were determined using Spearman’s rank correlation test. Univariate and multivariate logistic regression analyses were used to identify the main predictors of albuminuria in COPD patients. The significance of the results obtained was judged at the 5% level.

## Results

In the current study, the patient group included 60 adult male patients with COPD, with a mean age of 58.8 ± 8 years. The control group included 40 adult male subjects free of COPD, with a mean age of 57.23 ± 7.3 years. In Table 1, we compared the personal and anthropometric data of patients and controls. We also compared some physiological and laboratory parameters between the two groups. Patients with COPD had significantly lower oxygen saturation than non-COPD subjects (*p* < 0.001). The spirometric parameters, including FEV_1_ (%pred), FVC (%pred), FEV_1_/FVC%, PEF (%pred), and FEF_25−75%_ (%pred), were all significantly lower in the COPD patient group compared to the control subjects (*p* < 0.001 for each).

**Table 1:** Comparison between COPD patients and the control group according to personal, anthropometric, physiological, and laboratory data.

	Patients (*n* = 60)	Control (*n* = 40)	*P*
Age (years)	58.8 ± 8^a^	57.23 ± 7.3^a^	0.322
Residence: Urban/Rural (%)	55/5 (91.7)	36/4 (90)	^FE^*p*= 1.000
Smoking status			
Current, n (%)	50 (83.3)	31 (77.5)	0.466
Ex-smoker, n (%)	10 (16.7)	9 (22.5)	
Intensity (cigarettes/day)	20 (20–30)^b^	20 (18.8–30)^b^	0.497
Duration of smoking (years)	41 (32–48.5)^b^	39.5 (29.8–42)^b^	0.103
Smoking Pack-Year Index	44 (30.5–73.1)^b^	41.5 (27.4–55.5)^b^	0.162
Age at starting smoking (years)	15 (12–19.8)^b^	16.5 (15–20)^b^	0.112
Weight (kg)	74.2 ± 17.4^a^	80.2 ± 14.6^a^	0.075
Height (cm)	170.4 ± 7.9^a^	169.8 ± 10.8^a^	0.749
BMI (kg/m^2^)	25.5 ± 5.3^a^	27.1 ± 3.5^a^	0.096
Waist circumference (cm)	101.4 ± 15.2^a^	105.3 ± 10.0^a^	0.156
Systolic arterial blood pressure (mmHg)	120 ± 14.3^a^	122.5 ± 12.2^a^	0.367
Diastolic arterial blood pressure (mmHg)	80.2 ± 9.1^a^	81.1 ± 8.0^a^	0.613
SpO_2_%	94.7 ± 2.6^a^	97 ± 1.4^a^	< 0.001^*^
Fasting blood glucose (mg/dL)	99.6 ± 14.8^a^	95.2 ± 12.5^a^	0.102
Total cholesterol level (mg/dL)	187.0 ± 37.7^a^	191.2 ± 43.8^a^	0.610

a; mean ± standard deviation, b; median (interquartile range), *p*; p-value for comparison between the two studied groups, FE; Fisher Exact test, BMI; body mass index, SpO_2_; peripheral oxygen saturation, *; Statistically significant at *p* ≤ 0.05.

**Fig. 1:**
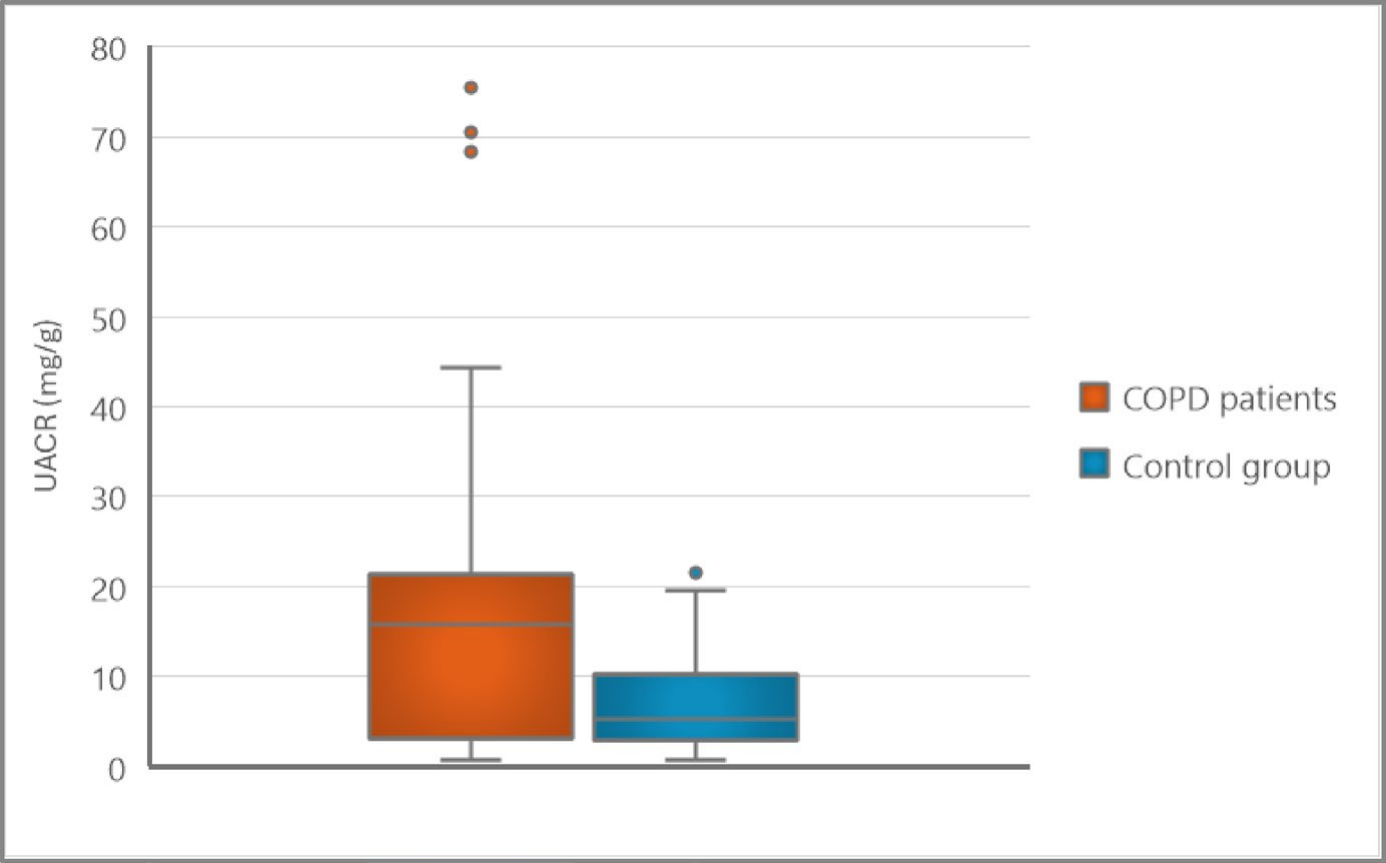
Boxplot showing UACR (mg/g) of COPD patients and the control group. The upper and lower borders of boxes represent the third and first quartiles, center lines denote the median, and whiskers show the minimum and maximum. UACR; urine albumin-to-creatinine ratio, mg; milligram, g; gram.

**Fig. 2:**
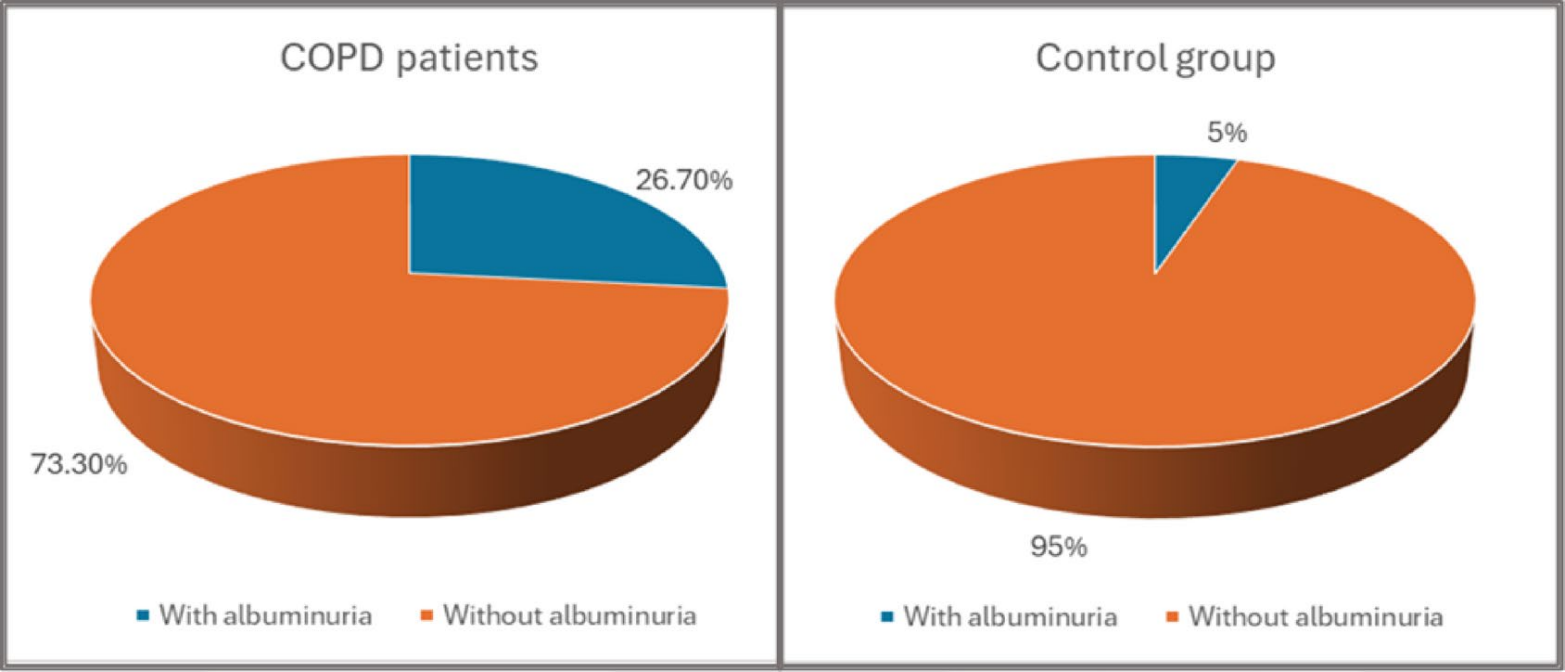
Pie charts showing the prevalence of albuminuria among COPD patients and the control group.

Comparing the UACR between the two groups, patients with COPD had significantly higher levels of UACR (mg/g) than the control group [15.8 (3.3–20.9) vs. 5.3 (2.9–10.1), *p* = 0.004], Fig. 1. The difference between groups persisted after the prevalence of albuminuria was compared using an accepted pathological threshold (UACR ≥ 20 mg/g)^19–21^ (26.7% of COPD patients vs. 5% of control subjects, *p* = 0.007), Fig. 2.

For further analysis, the group of patients with COPD was subdivided according to their UACR into two subgroups: patients with albuminuria, including those with UACR ≥ 20 mg/g, and patients without albuminuria, including those with UACR < 20 mg/g. The distribution of patients with COPD according to their UACR is presented in Table 2.

**Table 2:** Distribution of patients with COPD according to their UACR (*n* = 60).

	No. (%)	Mean ± SD.	Median (IQR)	Min.–Max.
Patients with albuminuria (UACR ≥ 20 mg/g)	16 (26.7)	42.7 ± 22.0	30.75 (24.2–69.5)	21.3–75.5
Patients without albuminuria (UACR < 20 mg/g)	44 (73.3)	9.6 ± 7.3	7.55 (2.7–16.6)	0.7–19.6
Total	60 (100)	18.2 ± 19.3	15.75 (3.3–20.9)	0.7–75.5

UACR: urine albumin-to-creatinine ratio, SD: standard deviation, IQR: interquartile range, min: minimum, max: maximum.

Comparing the personal and anthropometric data of COPD patients with and without albuminuria, patients with albuminuria had a significantly higher BMI (kg/m²) than those without albuminuria (*p* = 0.022). Regarding the GOLD ABE clinical classification of COPD, the ratio of patients with albuminuria who had frequent exacerbations (group E) was significantly higher than those without albuminuria (62.5% vs. 29.5%, *p* = 0.020). Table 3 presents the comparison between the two patient subgroups regarding their personal, anthropometric, and clinical characteristics.

**Table 3:** Comparison between COPD patients with and without albuminuria according to personal, anthropometric, and clinical data.

Total (*n* = 60)		Patients with albuminuria (*n* = 16)	Patients without albuminuria (*n* = 44)	*P*	
Age (years)		58.8 ± 8^a^	58.2 ± 6.5^a^	59 ± 8.6^a^	0.753
Residence (%Urban)		55 (91.7)	15 (93.8)	40 (90.9)	^FE^*p*=1.000
**Smoking status**					
Current, n (%)		50 (83.3)	12 (75.0)	38 (86.4)	^FE^*p*=0.433
Ex-smoker, n (%)		10 (16.7)	4 (25.0)	6 (13.6)	
Smoking Pack-Year Index		44 (30.5–73.1)^b^	47 (37.5–75.6)^b^	44 (30.5–71.3)^b^	0.832
BMI (kg/m^2^)		25.5 ± 5.3^a^	28.4 ± 6.2^a^	24.4 ± 4.6^a^	0.022^*^
Waist circumference (cm)		101.4 ± 15.2^a^	107.5 ± 20.8^a^	99.2 ± 12.4^a^	0.215
Duration of COPD (years)		10 (3–18)^b^	8 (3–22.5)^b^	10 (3–17)^b^	0.870
Dyspnea (mMRC)		2 (1–3)^b^	2.5 (1–4)^b^	1.5 (1–2.5)^b^	0.150
CAT		19.4 ± 9.2^a^	20.2 ± 8.9^a^	19.1 ± 9.4^a^	0.745
SGRQ		58.2 ± 23^a^	58.8 ± 23.5^a^	56.4 ± 22.5^a^	0.763
**ABE group, n (%)**					0.066
A		20 (33.3)	3 (18.8)	17 (38.6)	^FE^*p*=0.148
B		17 (28.3)	3 (18.8)	14 (31.8)	^FE^*p*=0.518
E		23 (38.3)	10 (62.5)	13 (29.5)	0.020^*^
**GOLD Grade, n (%)**					
Grade 1		13 (21.7)	4 (25)	9 (20.5)	^MC^*p*=0.962
Grade 2		28 (46.7)	7 (43.8)	21 (47.7)	
Grade 3		14 (23.3)	4 (25)	10 (22.7)	
Grade 4		5 (8.3)	1 (6.3)	4 (9.1)	
Charlson index		3 (2–4)^b^	3 (3–4)^b^	3 (2–4)^b^	0.173

a; mean ± standard deviation, b; median (interquartile range), *p*; p-value for comparison between the two studied groups, FE; Fisher Exact test, BMI; body mass index, mMRC: modified Medical Research Council, CAT: COPD assessment test, SGRQ: St. George’s Respiratory Questionnaire, MC: Monte Carlo, *; Statistically significant at *p* ≤ 0.05.

Regarding the physiological parameters, the results of the post-bronchodilator spirometry test revealed that the median (IQR) of the bronchodilator response (% change in FEV_1_ after SABA inhalation) was 6% (0–16.9%), Table 4. Although the mean post-bronchodilator FVC (%pred) was lower in patients with albuminuria than in those without albuminuria, the difference was not statistically significant (*p* = 0.489).

**Table 4:** Comparison between COPD patients with and without albuminuria according to physiological data.

	Total (*n* = 60)	Patients with albuminuria (*n* = 16)	Patients without albuminuria (*n* = 44)	*P*
Post-BD FEV_1_ (%pred)	59.9 ± 20.7^a^	58.6 ± 20.9^a^	60.3 ± 21^a^	0.802
Post-BD FVC (%pred)	79.4 ± 19.8^a^	75.9 ± 17.5^a^	80.6 ± 20.6^a^	0.489
Post-BD FEV_1_/FVC	59.6 (54.2–65.1)^b^	59.5 (54.2–63.8)^b^	62.9 (52.3–65.3)^b^	0.617
Bronchodilator response (% change in FEV_1_)	6 (0–17)^b^	5.6 (0.4–17.3)^b^	9.6 (0–17)^b^	0.980
Post-BD PEF (%pred)	50.8 ± 22.3^a^	50.5 ± 22.6^a^	51.4 ± 22.4^a^	0.916
Post-BD FEF_75%_ (%pred)	29.5 ± 15.6^a^	28.5 ± 12.8^a^	32.3 ± 22.2^a^	0.587
Post-BD FEF_25−75%_	31.4 ± 17.9^a^	30.5 ± 15.7^a^	34 ± 23.6^a^	0.567
HR (beat/min)	74.5 ± 10.1^a^	79 ± 10.7^a^	72.9 ± 9.6^a^	0.075
Systolic ABP (mm Hg)	120 ± 14.3^a^	123.3 ± 15.1^a^	118.8 ± 14.1^a^	0.288
Diastolic ABP (mm Hg)	80.2 ± 9.1^a^	82.3 ± 9.1^a^	79.4 ± 9.2^a^	0.283
SpO_2_%	94.70 ± 2.60^a^	91.25 ± 1.86^a^	95.91 ± 1.48^a^	< 0.001^*^
6MWD (m)	138.5 (120–190)^b^	138.5 (120–190)^b^	142 (128.5–181.5)^b^	0.802
BODE Index	4.9 ± 2.5^a^	4.5 ± 2^a^	5 ± 2.7^a^	0.563
FMD (% change in brachial artery diameter)	4.13 ± 1.23^a^	3.35 ± 0.94^a^	4.52 ± 1.38^a^	0.003*

a; mean ± standard deviation, b; median (interquartile range), p: p-value for comparing COPD patients with albuminuria and patients without albuminuria, post-BD: post-bronchodilator value, FEV_1_: forced expiratory volume in the first second, %pred: percent of predicted value, FVC: forced vital capacity, PEF: peak expiratory flow, FEF_50%_: forced expiratory flow at 50% of the FVC, FEF_75%_: forced expiratory flow at 75% of the FVC, FEF_25−75%_: forced expiratory flow at 25–75% of the FVC, HR: heart rate, RR: respiratory rate, ABP: arterial blood pressure, SpO_2_; peripheral oxygen saturation, 6MWD: six-minute walk distance, BODE index: (Body mass index, airflow Obstruction, Dyspnea, and Exercise capacity) index, FMD: flow-mediated dilation.

*: Statistically significant at *p* ≤ 0.05.

The same table also shows that patients with albuminuria had significantly lower oxygen saturation than those without albuminuria (*p* < 0.001). Simultaneously, they had significantly lower FMD of the brachial artery (*p* = 0.003).

Table 5 shows the main laboratory results of our COPD patients. In all patients (*n* = 60), the serum creatinine level (mg/dL) and estimated glomerular filtration rate (eGFR) (mL/min) were within the normal range (mean ± SD = 1 ± 0.2 and 94.3 ± 21.1, respectively). Although none of our patients were diagnosed with diabetes mellitus [mean fasting plasma glucose level (mg/dL) = 99.6 ± 14.8], patients with albuminuria showed a significantly higher fasting plasma glucose level (mg/dL) than patients without albuminuria (*p* < 0.005). Finally, the total serum cholesterol level (mg/dL) was higher in patients with albuminuria than in patients without albuminuria (198.8 ± 45.2 vs. 182.9 ± 34.6); however, this difference was not statistically significant (*p* = 0.214).

**Table 5:** Comparison between COPD patients with and without albuminuria according to laboratory data.

	Total (*n* = 60)	Patients with albuminuria (*n* = 16)	Patients without albuminuria (*n* = 44)	*P*
Serum creatinine	1 ± 0.2	1 ± 0.3	1 ± 0.2	0.707
eGFR (mL/min)	94.3 ± 21.1	98.7 ± 28	92.8 ± 18.4	0.416
Fasting plasma glucose (mg/dL)	99.6 ± 14.8	109.8 ± 13.5	95.9 ± 13.6	0.004^*^
Total serum cholesterol (mg/dL)	187 ± 37.7	198.8 ± 45.2	182.9 ± 34.6	0.214

p: p-value for comparing COPD patients with albuminuria and patients without albuminuria, eGFR: estimated glomerular filtration rate.

Data were presented as mean ± SD.

*: Statistically significant at *p* ≤ 0.05.

**Fig. 3:**
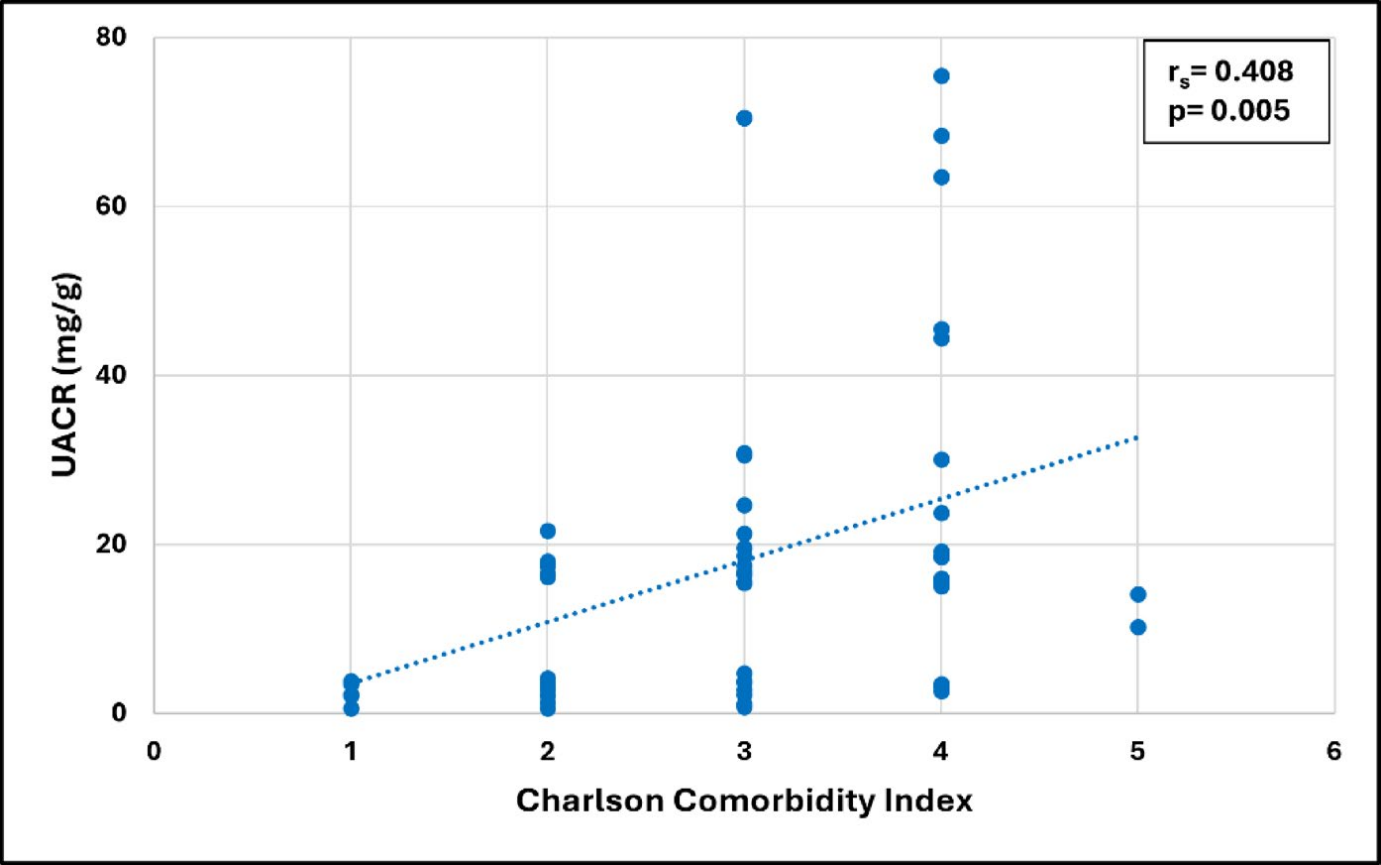
Correlation between UACR and Charlson Comorbidity Index in patients with COPD (*n* = 60).

**Fig. 4:**
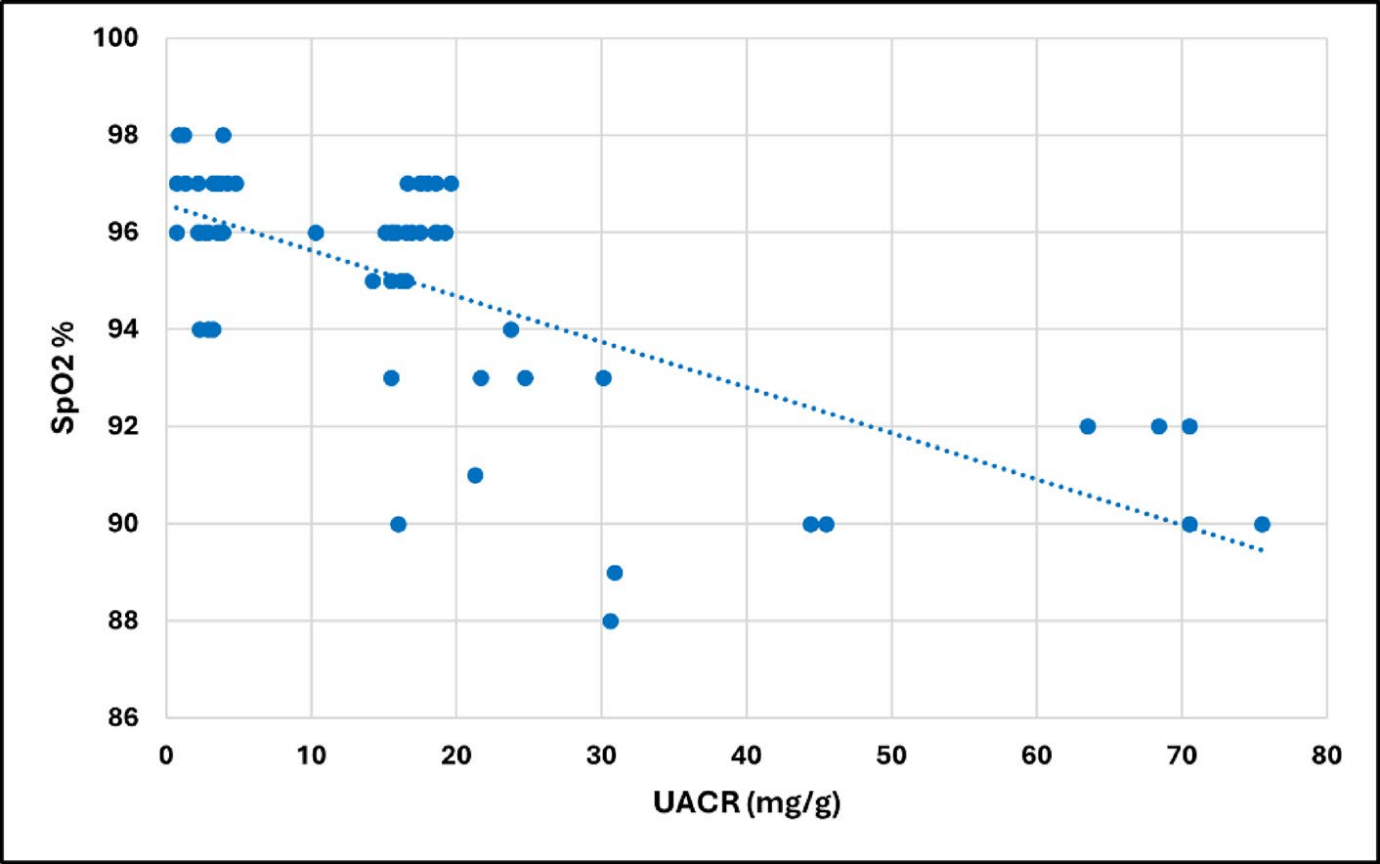
Correlation between UACR and SpO_2_ in patients with COPD (*n* = 60). SpO_2_; peripheral oxygen saturation

**Fig. 5:**
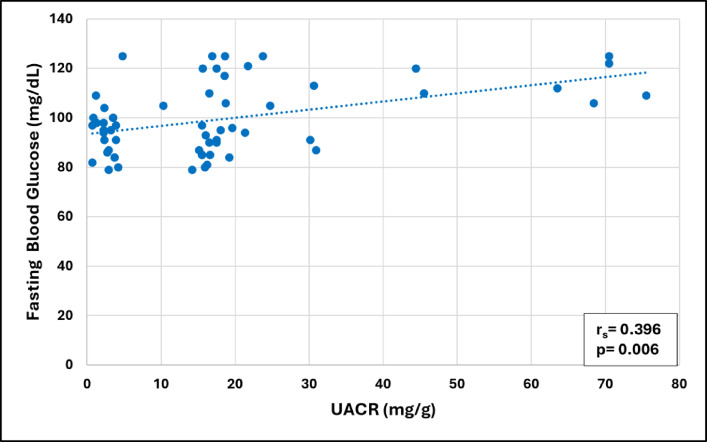
Correlation between UACR and fasting blood glucose level (mg/dL) in patients with COPD (*n* = 60).

For the whole group of COPD patients, the UACR had a significant positive association with the Charlson Comorbidity Index (CCI) (*p* = 0.005) and the fasting plasma glucose level (*p* = 0.006), and a significant negative association with the SpO_2_ (*p* < 0.001), as shown in Figs. 3, 4, and 5, respectively. Additionally, the UACR was negatively correlated to the FMD of the brachial artery, an indicator of generalized endothelial function (*p* = 0.042).

Univariate and multivariate logistic regression analyses showed that high BMI, low SpO_2_, and high fasting blood glucose level were the main predicting factors of albuminuria. Low oxygen saturation (SpO_2_) was the only independent predictor for albuminuria in patients with COPD (Table 6).

**Table 6:** Univariate and multivariate logistic regression analysis for the parameters affecting the presence of albuminuria in patients with COPD (*n* = 16 vs. 44).

Parameter	Univariate			^#^Multivariate		
	OR	95% CI	p	OR	95% CI	P
Age (years)	0.99	0.91–1.07	0.747			
Charlson index	1.62	0.79–3.33	0.188			
BMI	1.17	1.01–1.35	0.031^*^	1.208	0.83–1.76	0.324
Systolic ABP	1.02	0.98–1.07	0.348			
SpO_2_	0.31	0.15–0.62	0.001^*^	0.264	0.11–0.61	0.002^*^
6MWD	0.99	0.99–1.01	0.523			
Serum Creatinine	2.18	0.11–44.42	0.612			
Fasting Blood Glucose	1.07	1.02– 1.13	0.009^*^	1.109	0.98–1.25	0.091
HR	1.07	0.99–1.14	0.081			
ABE Classification [Group E]	2.57	0.67–9.86	0.170			

## Discussion

In this study, the evaluation of albuminuria in patients with COPD compared to non-COPD smokers revealed significantly higher levels of UACR (mg/g) among COPD patients. Albuminuria was significantly higher in COPD patients (26.7%) compared to control subjects (5%).

This was in accordance with Romundstad et al.^21^, who reported albuminuria in 21.6% of COPD patients vs. 7.5% of control subjects. However, the participants in their study were included regardless of self-reported hypertension and/or diabetes mellitus, which are known to have strong associations with albuminuria^22^.

Additionally, in our study, the demographic, anthropometric, and smoking data did not show any significant difference between the two groups. This was in contrast to Casanova et al.^19^, who recruited control subjects who were significantly younger and smoked less than the COPD patients. Many comorbid conditions have shared risk factors with COPD and are still not proven to be more common in COPD after adjusting for these risk factors. Thus, accurate analysis requires comparison with age- and smoking-matched control groups, even if difficult to recruit.

For further analysis, we subdivided the group of COPD patients according to their UACR into two subgroups: patients with albuminuria, including those with UACR ≥ 20 mg/g, and patients without albuminuria, including those with UACR < 20 mg/g. The main characteristics of the two subgroups were compared.

The first reported difference between the two subgroups was that patients with albuminuria had significantly higher BMI than those without albuminuria, and the BMI was one of the predicting factors of albuminuria among COPD patients. Many studies have reported that obesity leads to glomerular hyperfiltration, causing a higher GFR and increased renal blood flow, thereby contributing to kidney damage^23^. Additionally, there is considerable evidence that obesity is associated with ED through several mechanisms, including oxidative stress, insulin resistance (IR), and inflammation^24^. First, obesity is associated with the development of hypertension, hyperlipidemia, diabetes, and metabolic syndrome, all of which promote the production of reactive oxygen species (ROS) and induce ED^25^. In addition, obesity is a risk factor for the development of obstructive sleep apnea syndrome, which results in intermittent hypoxia and sleep fragmentation. Intermittent hypoxia, through different mechanisms, causes inflammation, IR, and oxidative stress and impairs endothelial function^26^. Finally, obesity is characterized by adipocyte dysfunction leading to an imbalance between the production of proinflammatory and anti-inflammatory adipokines, which also promotes IR and inflammation, leading to the development of ED and hence albuminuria^27^.

Interestingly, we did not find significant differences between the two subgroups of patients regarding their smoking data. This result extends the results of Ambrosino et al.^28^ and Hancox et al.^29^, who showed an association between COPD and ED, which was independent of the baseline smoking status. Also consistent with our findings, Bulcun et al.^20^ did not find a significant relationship between the presence of albuminuria and the smoking status of COPD patients.

Although cigarette smoke (CS) was regarded as the major common risk factor between COPD and ED, this statement is currently restrictive. A similar association has been recognized concerning exposure to other forms of inhaled particles, including atmospheric pollution, particularly in low- and middle-income countries (LMICs)^30^. With increasing research concerning COPD in LMICs, only 30–40% of COPD in this setting is attributed to CS, and thus, non-CS environmental factors are of greater significance^31^. This may be specifically applicable to our patients, as 92% of them were living in urban areas, most were of low socioeconomic status, and had high workplace exposure levels. Similar to CS^2^, the association between air pollution and ED includes direct damage to the endothelial cells (ECs) and indirect mechanisms like oxidative stress, systemic inflammation, and impaired nitric oxide (NO) bioavailability, especially on the microvascular level^32^.

The clinical classification of our patients using the GOLD^8^ ABE tool showed 62.5% of patients with albuminuria vs. 29.5% of patients without albuminuria to be frequent exacerbators (group E), and the difference was statistically significant (*p* = 0.020). In their 1-year follow-up study, Bartziokas et al.^33^ also reported albuminuria in COPD patients as an independent predictor of acute exacerbation. Acute exacerbation is an important feature in the natural history of the disease and is associated with worse outcomes. Therefore, the fact that albuminuria is associated with more frequent/more severe exacerbations may suggest a possible role for this biomarker in the identification of exacerbation-prone patients.

Another agreement between our results and Bartziokas et al.^33^ was the presence of a positive association between the UACR and the Charlson comorbidity index (CCI). The comorbidities affecting patients with COPD have been suggested to result from syndemics, defined as the occurrence of disease clusters with shared risk factors and pathobiological mechanisms that negatively affect the prognosis and burden of each disease^34^. This conceptual framework should advance the strategies for disease management by focusing on the development of biomarkers for the assessment of these pathobiological mechanisms. Using albuminuria as a marker of systemic disease burden could broaden its clinical relevance in this patient population. In particular, the possible relationship between albuminuria and frailty-related cardiovascular comorbidities which may be overlooked because of overlapping clinical symptoms and signs^35^.

Among the measured spirometric parameters in our study, the FVC was more reduced in patients with albuminuria; however, it did not reach the level of statistical significance. The reduction in FVC might be explained by the lung hyperinflation that has been previously linked to ED in COPD patients^36^. Nevertheless, among our patients, we did not find significant differences regarding the spirometric parameters related to small airway function: FEF_75%_ and FEF_25−75%_. In 2019, Kang et al.^37^ investigated a group of 3345 COPD patients and reported that patients with low FVC had a higher BMI, higher waist circumference, and more metabolic comorbidities compared to those with normal FVC. They proposed the presence of a distinctive metabolic phenotype among COPD patients characterized by low FVC, obesity, and metabolic comorbidities, which was later suggested by other studies^38^. In line with these results, our patients with albuminuria have also shown significantly higher BMI and higher fasting blood sugar levels. Despite not reaching the level of statistical significance, they also had lower FVC, higher waist circumference, higher systolic and diastolic arterial blood pressure, and higher serum cholesterol levels than those without albuminuria.

In the current study, patients with albuminuria also had significantly lower FMD of the brachial artery than those without albuminuria, and the UACR was negatively correlated with the percentage change in the brachial artery diameter. A similar association was reported in two recent studies^39,40^ conducted on diabetic patients. The same findings were reported in nondiabetic individuals^41,42^, supporting the possible use of albuminuria for early detection of ED in patients at risk.

In our study, the most significant difference between the two subgroups of COPD patients was oxygen saturation (SpO_2_), assessed by pulse oximetry. Patients with albuminuria had significantly lower levels of SpO_2_, indicating more hypoxemia, than those without albuminuria. SpO_2_ had a strong negative association with UACR and was the only independent predictor of albuminuria in patients with COPD.

The observed decrease in SpO_2_ likely reflects impaired lung diffusing capacity, which has been negatively correlated with albuminuria in previous studies^43,44^. This may imply an important pathophysiological link between the former, representing pulmonary microangiopathy, and the latter, representing extrapulmonary microangiopathy. On the other hand, a causal relationship was suggested by Laursen et al.^45^, where hypoxemia could be a driver of multiple organ dysfunction, leading to various complications, including albuminuria. Hypoxia can cause disturbances to vascular homeostasis and ED and is believed to contribute to the pathophysiology of CVD in COPD patients. The development of CVD in patients with COPD will further exacerbate vascular hypoxia, resulting in a positive feedback loop^46^.

The finding that albuminuria was significantly associated with SpO_2_ but not with 6MWD may reflect distinct underlying pathophysiological mechanisms. While albuminuria may reflect microvascular dysfunction or impaired gas exchange at rest, the 6MWD is influenced by various factors, including skeletal muscle performance, cardiopulmonary reserve, and psychological motivation. Previous studies also focused on the contribution of COPD comorbidities, such as CVD and mood disorders, to exercise limitation and 6MWD^47^. The present results, as well, confirm the complexity of 6MWT determinants in COPD.

Finally, our study had a number of limitations. No female participants were included, and this made it impossible to evaluate possible sex differences in our findings. However, the absence of females was not by design, because we offered the opportunity to join the study independent of sex. It is also possible that the sample size may not allow the detection of some significant differences between patients with and without albuminuria (type II error). In addition, we could have assessed various biomarkers proposed to be involved in the pathophysiological link between COPD and ED. However, this was beyond the objectives of the current study and may be addressed in future research.

However, to our knowledge, this is the first study to assess the presence of albuminuria in a group of COPD patients in comparison to a group of age- and sex-matched control subjects with an equivalent smoking history to eliminate the possible effect of shared risk factors. For the same reason, we also excluded subjects with hypertension and/or diabetes mellitus due to their known association with ED. In addition to UACR, we assessed the FMD of the brachial artery, the gold standard measure of endothelial function, to validate our findings. Finally, to investigate the relationship between albuminuria and the physiological and clinical outcomes of COPD, we evaluated a wide range of variables, all of which were simple and non-invasive, so that they could be assessed in the outpatient clinic, as indicated in the aim of our study.

In conclusion, our study supports the hypothesis that albuminuria is more prevalent in patients with stable COPD than in non-COPD smokers. The presence of albuminuria in COPD patients may be used as an indicator of the presence of ED, given the negative association reported between UACR and FMD of the brachial artery, the gold standard measure of ED. The UACR in COPD patients is also negatively associated with O_2_ saturation and positively associated with the number of comorbidities and fasting plasma glucose level. The predictors of albuminuria in COPD patients are high BMI, low oxygen saturation, and high fasting plasma glucose levels. However, the only independent predictor is the presence of low oxygen saturation. Thus, measurement of UACR could be routinely utilized as a simple, inexpensive, and noninvasive method for assessment of ED in COPD patients, especially in those with low O_2_ saturation.

## Data Availability

The datasets that support the current study are available from the corresponding author upon reasonable request.
